# Thick bark can protect trees from a severe ambrosia beetle attack

**DOI:** 10.7717/peerj.10755

**Published:** 2021-02-16

**Authors:** John M. Boland, Deborah L. Woodward

**Affiliations:** 1 Boland Ecological Services, San Diego, CA, United States of America; 2 California Water Quality Control Board, San Diego Region, San Diego, CA, United States of America

**Keywords:** *Euwallacea kuroshio*, Goodding’s black willow, Kuroshio shot hole borer, Refuge in size, Spatial pattern, *Salix gooddingii*, Tijuana River Valley

## Abstract

Thick bark has been shown to protect trees from wildfires, but can it protect trees from an ambrosia beetle attack? We addressed this question by examining the distribution of holes of the invasive Kuroshio Shot Hole Borer (KSHB, *Euwallacea kuroshio*; Coleoptera: Scolytinae) in the bark of Goodding’s black willow (*Salix gooddingii*), one of the KSHB’s most-preferred hosts. The study was conducted in the Tijuana River Valley, California, in 2016–17, during the peak of the KSHB infestation there. Using detailed measurements of bark samples cut from 27 infested trees, we tested and found support for two related hypotheses: (1) bark thickness influences KSHB attack densities and attack locations, i.e., the KSHB bores abundantly through thin bark and avoids boring through thick bark; and (2) bark thickness influences KSHB impacts, i.e., the KSHB causes more damage to thinner-barked trees than to thicker-barked trees. Our results indicate that thick bark protects trees because it limits the density of KSHB entry points and thereby limits internal structural damage to low, survivable levels. This is the first study to identify bark thickness as a factor that influences the density of KSHB—or any ambrosia beetle—in its host tree, and the first to link bark thickness to rates of host tree mortality.

## Introduction

The Kuroshio Shot Hole Borer (KSHB; *Euwallacea kuroshio* Gomez & Hulcr; Coleoptera: Scolytinae) is an ambrosia beetle native to Asia that has recently invaded southern California (Gomez et al., 2018). Unlike most ambrosia beetles, which infest dead wood (Kirkendall, Biedermann & Jordal, 2015), the KSHB attacks live trees. Before 2015, it had been found only in a few avocado groves and landscape trees (University of California Agriculture and Natural Resources, 2020), but in 2015 it became abundant in the native willow forests (*Salix* spp.) of the Tijuana River Valley (Boland, 2016). Over the next five-years, the KSHB population in the valley went through a boom-and-bust cycle and caused extensive damage to some of the willow forests, infesting an estimated 350,000 willows and killing an estimated 123,000 willows (Boland & Uyeda, 2020). There was a notable spatial pattern to the KSHB impact in the valley; rates of infestation, damage, and mortality were significantly higher in willows near the main river channel than in those farther away, even though all sites were dominated by the same two willow species (Boland, 2016; Boland & Woodward, 2019). The Tijuana River is frequently polluted with sewage, an effective plant fertilizer, and the spatial pattern in KSHB impact was hypothesized to be due to differences in the nutrient condition of the willows and a KSHB preference for fast-growing, nutrient-enriched trees that have wood of low density and high moisture content, i.e., the nutrient enriched condition of trees near the river made them more susceptible to KSHB impacts (Enriched Tree Hypothesis; Boland & Woodward, 2019).

Here we explore another condition of the willows—bark thickness—and ask whether willows growing farther from the river were less susceptible to the KSHB because of their thicker bark. In general, bark is known to protect trees from various external threats; it can insulate trees from wildfire, sun scorch and frost, and can defend against destructive mammals, insects, birds, bacteria and fungi (Vines, 1968; Franceschi et al., 2005; Midgley, Lawes & Chamaille-Jammes, 2010; Lawes et al., 2011; Wojtech, 2011; Ferrenberg & Mitton, 2014). Secondary chemical compounds in bark play an important protective role; resins, for example, are expelled when there is an injury to the bark and, because resins are toxic and harden when exposed to air, they seal wounds and deter many insects, e.g., bark beetles (Franceschi et al., 2005). In pines, bark texture has been shown to influence the locations of bark beetle attacks, with more attacks found on rough than on smooth bark surfaces (Ferrenberg & Mitton, 2014). As for bark thickness, thick bark has been shown to protect trees from wildfires (e.g., Vines, 1968; Lawes et al., 2011), and tree species in fire-prone areas have relatively thicker bark than those outside such areas (Schafer et al., 2015), but to our knowledge there are no published accounts of thick bark protecting trees from boring insect pests.

We predicted that thick bark would hinder KSHB infestation and tested two related hypotheses: (1) **Bark thickness influences KSHB attack densities and attack locations**, i.e., the KSHB bores abundantly through thin bark and avoids boring through thick bark; and (2) **Bark thickness influences KSHB impacts**, i.e., the KSHB causes more damage to thinner-barked trees than to thicker-barked trees. We tested these hypotheses by examining the distribution of KSHB holes in the bark of *Salix gooddingii* C.R. Ball (Goodding’s black willow, Salicaceae), a common native riparian tree and one of the most-preferred hosts of the KSHB (Boland, 2016; Coleman et al., 2019).

## Materials & Methods

### Study Site and Study Organisms

The study was conducted in the Tijuana River Valley, a small (14.6 square kilometer) coastal floodplain in San Diego County, California, at the end of a 4,480 square kilometer, binational watershed (Safran et al., 2017). The river is an intermittent stream that typically flows strongly in winter and spring and is mostly dry in summer (Boland, 2014). Because of frequent cross-border inputs of sewage, it is one of the most polluted rivers in the state (Gersberg, Daft & Yorkey, 2004; Boland & Woodward, 2019). The riparian forests in the valley are preserved within three adjoining open space parks, and two willow species, *S. gooddingii* and *S. lasiolepis* Benth., are numerically and structurally dominant (Boland, 2014). In 2015, when the KSHB was first detected in the valley, the riparian habitats were divided into 29 units for census purposes such that each unit was relatively homogenous in terms of species composition, willow age and willow density (Boland, 2016), and those units were retained for this study. The units can be grouped into Wet Forests, where the trees are flooded each winter, and Dry Forests, where the trees rarely receive river flows. The Dry Forests are dry because the river has migrated away from them. All of the Wet Forests were initially heavily damaged by the KSHB (Boland, 2016) but since 2016 they have substantially recovered and in 2020 they were similar to their pre-KSHB stature (Boland & Uyeda, 2020; Boland et al., 2020).

Female KSHB beetles bore through the bark and create their galleries inside the sapwood (xylem) of live trees. They carry with them the symbiotic fungi, *Fusarium kuroshium* and *Graphium kuroshium*, which they farm in the gallery walls and consume (Na et al., 2018). The closely related and invasive Polyphagus Shot Hole Borer, *Euwallacea whitfordiodendrus* (Schedl), also attacks live trees in southern California, and the damage caused by it and the KSHB is being called Fusarium Dieback (Umeda, Eskalen & Paine, 2016; California Department of Fish Wildlife, 2020). Specimens from the Tijuana River Valley were collected and identified as KSHB by Akif Eskalen at University of California Riverside and are stored in their collection. The County of San Diego Department of Parks and Recreation and the US Fish and Wildlife Service allowed access to their properties via permits ROE08.04.16 and 19001TJS, respectively.

### Bark samples

Bark samples were collected from 27 *S. gooddingii* trees in 2016–17, during the peak of the KSHB infestation in the valley (Boland & Uyeda, 2020). The sampled trees ranged from heavily infested and nearly dead to lightly infested and seemingly unaffected. They were from nine forest units where the stands were 0 m to 520 m from the river and five to 35 years old (Boland, 2016; Boland & Woodward, 2019). Of the nine forest units, five were Wet Forests (Units 2, 3, 9, 12 and 13) and four were Dry Forests (Units 15, 17, 19 and 21). In each unit, three infested trees were randomly chosen and, at each tree, the trunk diameter was measured and a bark sample was collected at breast height (1.37 m). Each bark sample was approximately 11 × 20 cm and cut using a hand saw, hammer, and chisel. The samples included inner and outer bark layers and no xylem. The scar was sprayed with TreeKote Tree Wound Dressing to reduce the risk of infection, and tools were cleaned between samples with a disinfectant wet wipe.

In the lab and within hours of collection, a 10 × 15 cm grid was drawn on the inner surface of each bark sample using a ruler and permanent marker, and the following counts and measurements were taken under a 3.5x desk magnifier: (1) the number of KSHB holes in the grid (# holes per 150 cm^2^); (2) the bark thickness at each hole (*n* = 0 to 113 holes per sample); and (3) the bark thickness at each grid line intersection (*n* = 150 points per sample). In total, 914 bark thickness measurements were made at KSHB holes and 4,050 bark thickness measurements were made at grid line intersections; these represented the thicknesses *used* by the KSHB and the thicknesses *available* to the KSHB, respectively. All bark thicknesses were measured using digital vernier calipers (Vinca DCLA-0605) fitted with extensions (Anytime Tools 4 Points Caliper Extension Set for Deep Measuring), allowing measurements at all points on the sample. Extension tips were two mm in diameter, small enough to measure in narrow furrows but not so small as to extend into the KSHB holes, which are approximately one mm in diameter. All bark thickness measurements were recorded to three decimal places (0.000 cm) and are presented to two decimal places. Precision was quantified by measuring the 150 grid line intersections on one bark sample twice and comparing the two sets of measurements; the Root Mean Square Error of the paired measurements was 0.07, the difference in median thickness was 0.014 cm, and the difference in mean thickness was 0.002 cm, indicating good precision.

Our use of relatively large bark samples, which included several of the vertical ridges and furrows characteristic of *S. gooddingii* bark, allowed us to document the range of bark thicknesses available to and used by the KSHB and to accurately count KSHB holes from the inner surface of the bark. Holes are often visible on the outer surface of the bark but are more reliably counted on the inner surface when bark is textured. Lenticels are areas of loosely packed cells in bark that allow for gas exchange, and potentially could be vulnerable spots for entry of boring insects (Rosner & Führer, 2002); however, lenticels in *S. gooddingii* are inconspicuous and small relative to KSHB holes, so we were unable to ascertain the use of lenticels as points of entry by KSHB. Throughout this paper, the term ‘holes’ refers to the bore holes that KSHB make straight through the bark into and out of their galleries in the xylem; the term ‘furrows’ refers to the cracks, crevices, grooves or fissures where bark is relatively thin; and the term ‘ridges’ refers to the ribs or spurs where bark is relatively thick (Vaucher & Eckenwalder, 2003; Wojtech, 2011).

### Bark thickness influences KSHB attack densities and attack locations (H1)

To test this hypothesis, the bark sample data were used in three ways. First, the relationship between the density of KSHB holes in a sample and the median bark thickness of the sample was examined using exponential regression. Because there were some hole densities of zero, a value of 0.1 holes was added to all densities to obtain the exponential best-fit line. Second, to test whether bark thickness influenced KSHB attack locations, the mean of all the bark thicknesses *used* (*n* = 914) was compared to the mean of all the bark thicknesses *available* (*n* = 4,050) using a *t*-test. Third, to test whether KSHB showed a preference for certain bark thicknesses, the similarity between bark thicknesses *used* and *available* was determined for each bark sample. A measure of similarity, the proportional similarity index (PSI; Zaret & Smith, 1984), was calculated for each bark sample. The PSI is:

*PSI =* ∑* min (p*_*i*_*, q*_*i*_*)*

where p_i_ and q_i_ are the proportion *available* (p) and proportion *used* (q) for each thickness increment (i), using 0.1 cm increments over the range <0.2 to 2.0 cm. In short, the PSI was a measure of how similar the frequency distributions of bark thicknesses *available* and *used* were for each bark sample. A high PSI meant a high similarity, or high overlap, in the frequency distributions and indicated the random choice of bark thickness by the KSHB. A low PSI meant a low similarity, or low overlap, in the frequency distributions and indicated a strong deviation from random choice of bark thickness by the KSHB. Exponential regression was used to test for significant correlation between the PSI and the median bark thickness.

### Bark thickness influences KSHB impacts (H2)

To test this hypothesis, the bark sample data were used with other data in four ways. First, the relationship of bark thickness to trunk diameter was examined to confirm that the expected relationship of bark thickness increasing with tree size (Rosell, 2016) occurs in *S. gooddingii*. Second, the relationship of KSHB hole density to trunk diameter was examined. Because there were some hole densities of zero, a value of 0.1 holes was added to all densities to obtain the exponential best-fit line. Third, the relationship of bark thickness to willow mortality rate was examined. Willow mortality rates for each unit were the maximum percent mortality obtained from three annual surveys of living and dead willows conducted in each unit between 2015 and 2018 (Boland, 2019). Fourth, to better understand the marked spatial pattern in the KSHB impacts in the valley, i.e., that willow mortality rates were significantly higher near the main river channel (Boland & Woodward, 2019), we compared the mean bark thicknesses of the samples from the Wet and Dry Forest units using a *t*-test.

### Comparison of two fallen trees

Two *S. gooddingii* trees that had fallen during 2017 provided an opportunity to estimate the total impact of KSHB on trees of different sizes. Both trees had been infested by KSHB while they were alive and standing, both had been blown down by strong winds, and both were examined soon after they fell. The smaller tree had broken at 1.4 m on the trunk (wind-snapped) due to extensive KSHB tunneling that undermined the structural integrity of the wood, whereas the larger tree had been uprooted (wind-thrown) with its trunk intact. The smaller tree (11 m tall and 10.8 cm diameter at breast height) was in a Wet Forest (Unit 2) in the current river bed, and the larger tree (23 m tall and 40.7 cm diameter at breast height) was in a Dry Forest 60 m from the current river bed (Unit 19; unit locations in Boland, 2016). Along the full trunk length of each tree, 1 m sections were marked and three measurements were taken within each section: maximum bark thickness, density of KSHB holes, and trunk circumference. Maximum bark thickness was measured in a bark sample (approximately 8 × 5 cm) cut with a hand-saw, hammer, and chisel such that the sample included inner and outer bark layers and no xylem; maximum thickness was measured using a pair of digital vernier calipers (Vinca DCLA-0605). KSHB holes were counted within a flexible quadrat (20 × 2 cm = 40 cm^2^) placed parallel to the axis of the trunk, and trunk circumference was measured with a tape.

To estimate the KSHB impact on the two trees, the volume of xylem damaged by KSHB and its associated fungi was estimated for each section of tree trunk. This estimate was done by assuming that each KSHB hole in the bark represented a 2.5 cm^3^ impact to the xylem. For each 1 m section of trunk, the total volume was calculated as a cylinder from the circumference measurement, the total number of KSHB holes was extrapolated from the density in the quadrat, the KSHB-impacted volume was calculated from the total number of holes multiplied by 2.5 cm^3^, and the KSHB-impacted volume was expressed as a percent of the total volume of the section. The assumption of a 2.5 cm^3^ impact per hole is based on measurements of many KSHB tunnel lengths (113 mm tunnel length per hole on average) and many fungal-induced cell deformities visible around KSHB tunnels (ellipse with a major radius of 4.7 mm and a minor radius of 1.5 mm on average; Boland unpublished data). This is a relatively conservative estimate of impact because others have found more extensive fungal impacts around the tunnels of other ambrosia beetles (Takahashi, Matsushita & Hogetsu, 2010).

## Results

### Bark samples

Overall, measurements of the bark thicknesses *available* ranged from 0.12 cm to 2.74 cm (*n* = 4, 050) and measurements of the bark thicknesses *used* ranged from 0.12 cm to 1.07 cm (*n* = 914). Among the individual bark samples, median thicknesses *available* ranged from 0.26 cm to 1.89 cm (*n* = 27) and KSHB hole densities ranged from 0 to 113 holes per 150 cm^2^ (Table 1). Seven samples had no holes, indicating that the trees from which they were taken were only lightly infested and holes were rare.

**Table 1:** Summary of bark sample measurements. Trunk diameters, bark thicknesses (*available*, used and Proportional Similarity Index (PSI)), and KSHB hole densities for each of the 27 bark samples. The samples are arranged in order of median bark thickness *available*. An ‘x’ indicates that a median bark thickness used could not be calculated because the bark sample had no KSHB holes.

SAMPLE #	TREE SIZE	BARK THICKNESS (cm)			KSHB HOLES
	diameter (cm)	AVAILABLE (median)	USED (median)	PSI (%)	density (# per 150 cm^2^)
1	8.9	0.26	0.27	94%	47
2	10.8	0.38	0.25	39%	80
3	16.6	0.46	0.45	89%	79
4	23.6	0.55	0.37	55%	49
5	25.1	0.56	0.32	31%	30
6	14.3	0.61	0.41	46%	54
7	17.8	0.61	0.43	46%	67
8	20.5	0.65	0.38	33%	21
9	24.2	0.68	0.59	74%	23
10	24.8	0.73	0.68	89%	113
11	25.1	0.76	0.38	42%	51
12	19.4	0.78	0.68	51%	63
13	22.9	0.81	0.72	73%	59
14	19.7	0.86	x	0%	0
15	28.3	0.88	0.53	35%	19
16	19.4	0.89	0.69	44%	51
17	35.0	0.97	0.76	52%	83
18	27.4	1.11	0.55	4%	2
19	22.6	1.21	0.72	25%	8
20	21.6	1.36	0.45	9%	14
21	28.3	1.42	x	0%	0
22	31.8	1.49	x	0%	0
23	30.6	1.51	x	0%	0
24	31.8	1.58	0.93	3%	1
25	34.7	1.58	x	0%	0
26	38.8	1.75	x	0%	0
27	39.2	1.89	x	0%	0

### Bark thickness influences KSHB attack densities and attack locations (H1)

**Figure 1:**
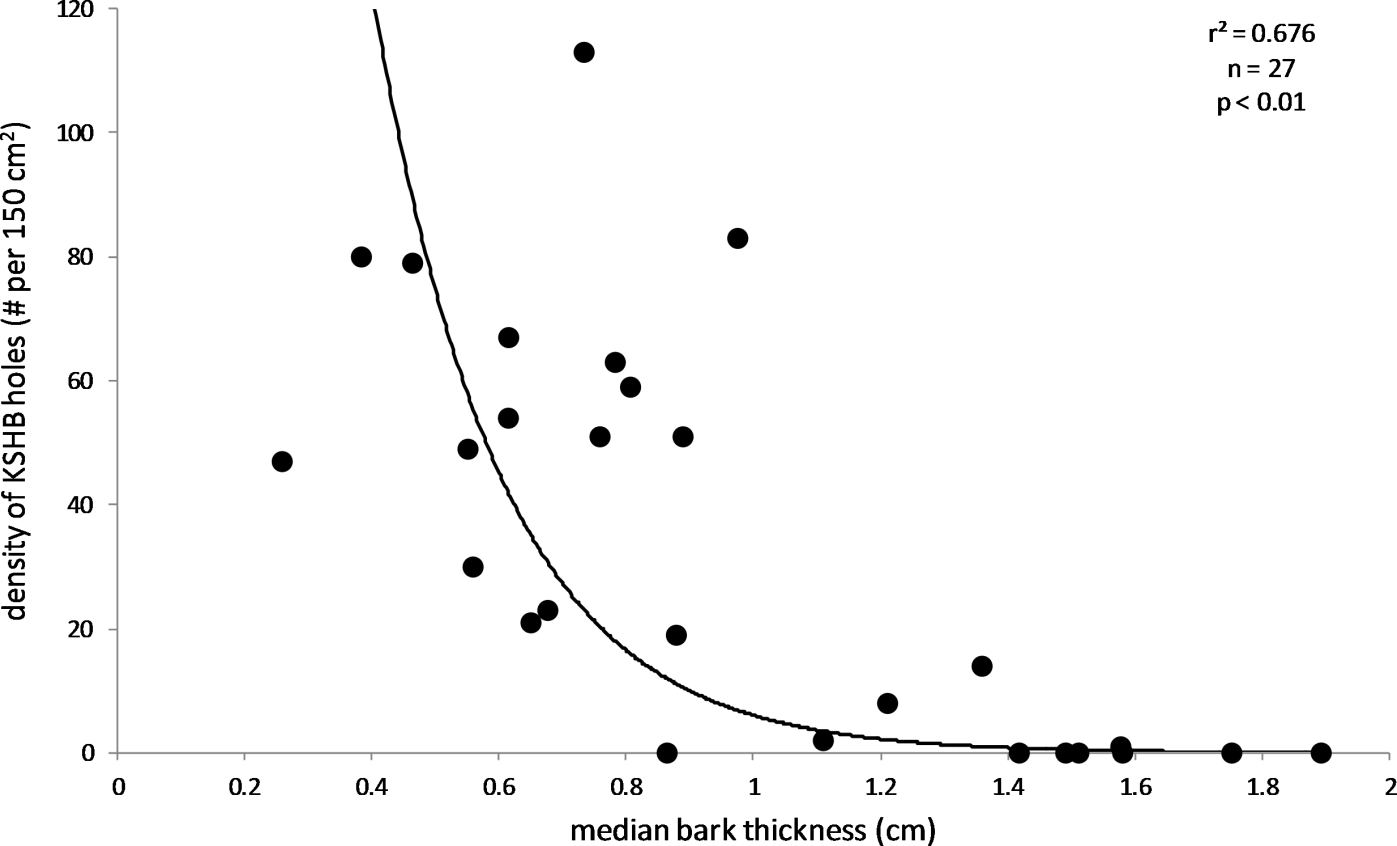
Influence of bark thickness on the density of KSHB holes. Median bark thicknesses and hole densities are from the 27 bark samples. The line of best fit and values of p and *r*^2^ are from exponential regression (*r*^2^ = 0.676; *p* < 0.01; *n* = 27).

**Figure 2:**
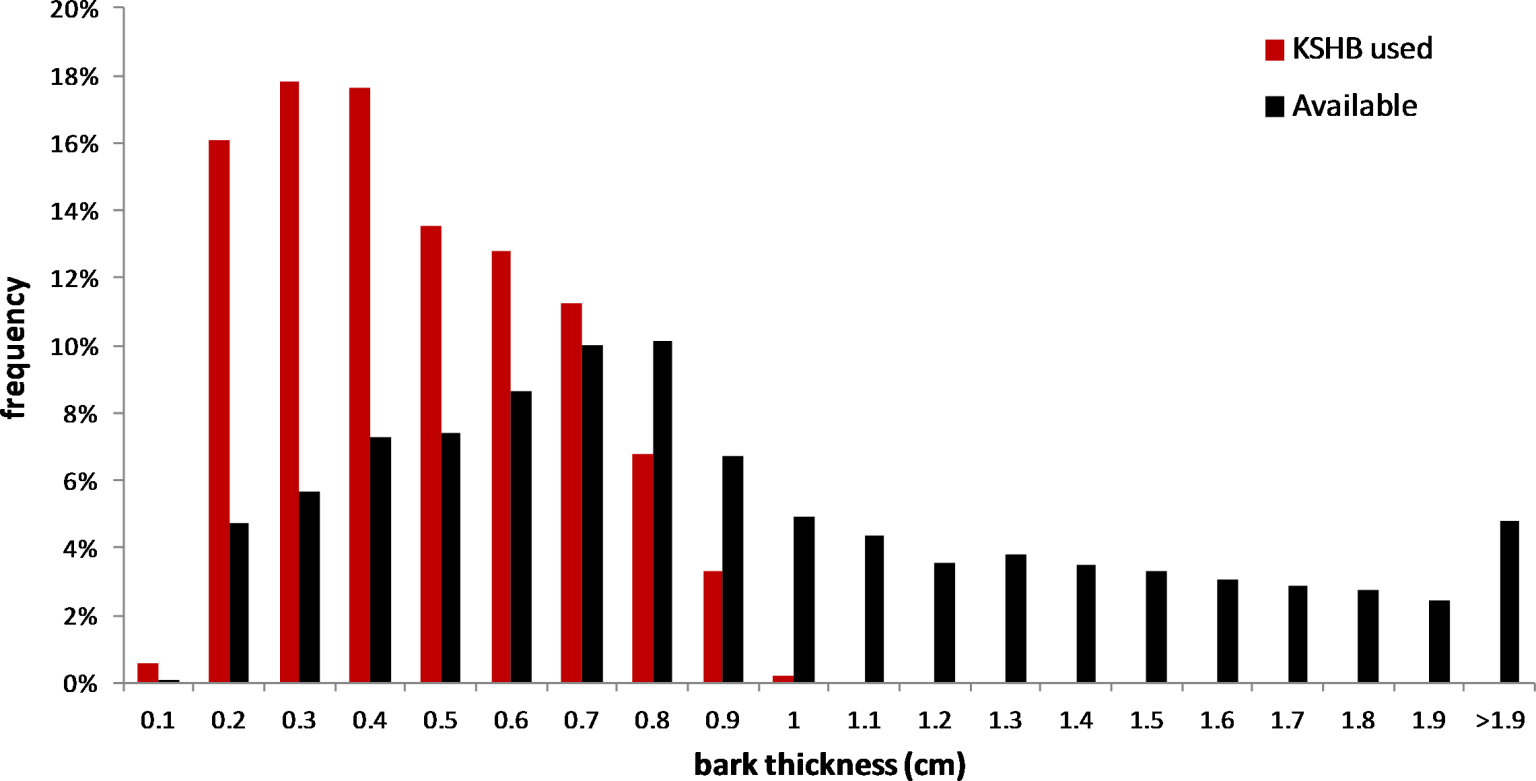
Overall bark thicknesses *used* and *available*. Frequency distributions of bark thicknesses *used* (measured at KSHB holes; *n* = 914) and bark thicknesses *available* (measured at grid line intersections, *n* = 4,050) in the 27 bark samples.

**Figure 3:**
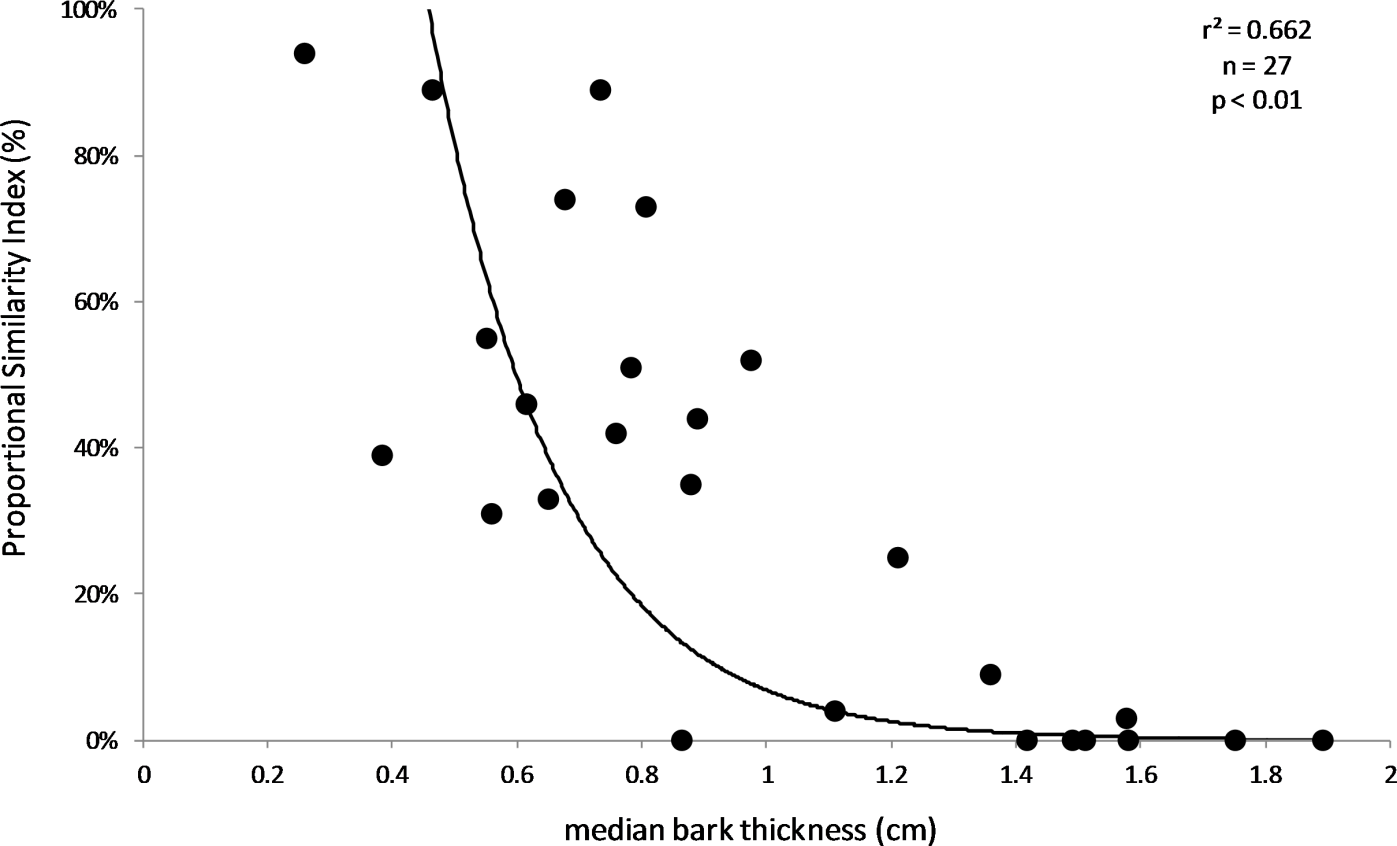
Relationship between Proportional Similarity Index (PSI) and bark thickness. PSI is a measure of overlap between the bark thicknesses *used* by and *available* to KSHB on each sample. The line of best fit was produced by exponential regression (*r*^2^ = 0.662; *p* < 0.01; *n* = 27).

The results supported the hypothesis that bark thickness influences KSHB attack densities and attack locations. First, hole density and median bark thickness were significantly and negatively correlated, indicating that thicker bark samples had significantly fewer holes (*r*^2^ = 0.676, *n* = 27, *p* < 0.01; Fig. 1). Second, the mean bark thickness *used* (0.52 ± 0.20 cm; *n* = 914) was significantly less than the mean bark thickness *available* (0.97 ± 0.54 cm; *n* = 4,050; *t*-test, *t* = 25.06, *p* < 0.01; Fig. 2), indicating that the KSHB did not use bark thicknesses in proportion to those available. KSHB bored into relatively thin bark (<0.8 cm) at a higher frequently than its availability and bored into relatively thick bark (>0.8 cm) at a lower frequently than its availability. No KSHB holes were in bark thicker than 1.07 cm, even though thicker bark was abundantly available. Third, the PSI declined significantly with increasing bark thickness, indicating that similarity between the thicknesses *used* and *available* decreased with bark thickness (*r*^2^ = 0.662, *n* = 27, *p* < 0.01; Fig. 3). In summary, bark samples that were generally thin had many KSHB holes and these holes were spread in a random distribution (high similarity between thicknesses *used* and *available*), whereas bark samples that were generally thick had few KSHB holes and these holes were found almost exclusively in furrows in a non-random distribution (low similarity between thicknesses *used* and *available*).

**Figure 4:**
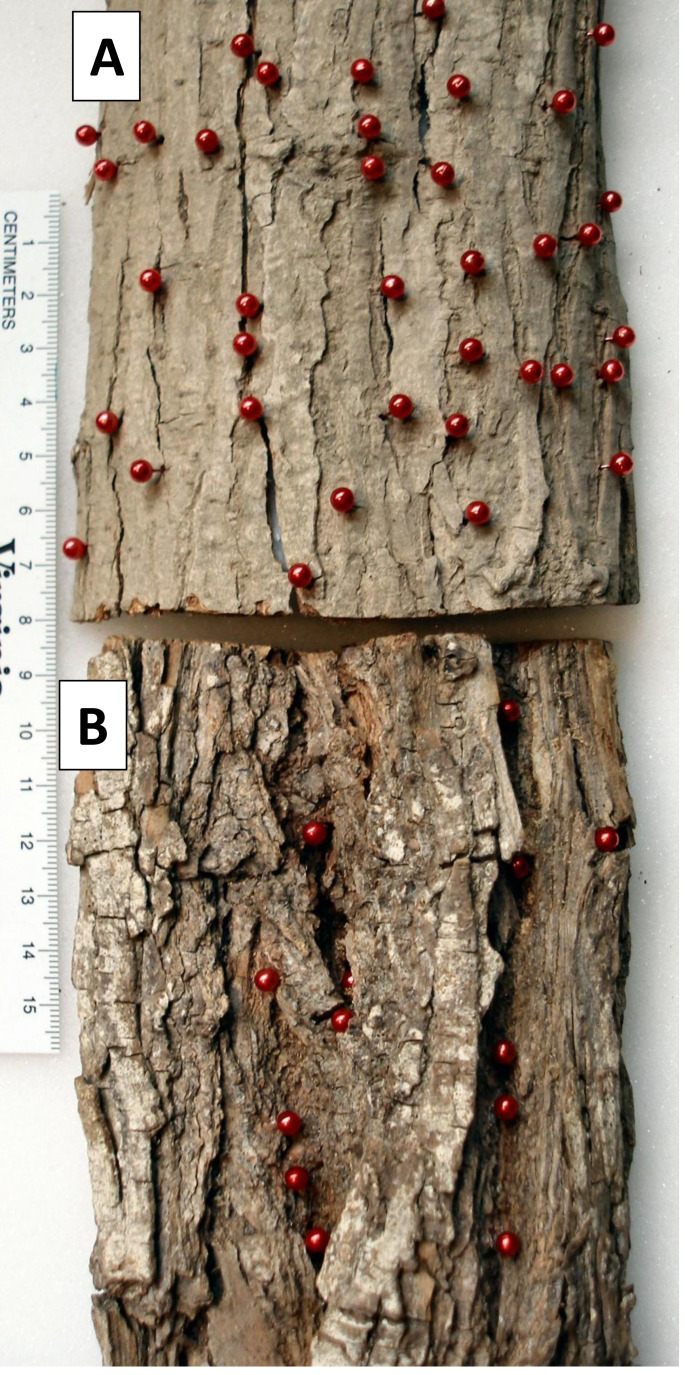
Comparison of two bark samples: view of outer surface. (A) The thin-barked sample #1 had 47 holes per 150 cm^2^. (B) The thick-barked sample #20 had 14 holes per 150 cm^2^. Most but not all of each sample is shown. KSHB holes in the bark samples are marked with red pins. Photo credit: John Boland.

**Figure 5:**
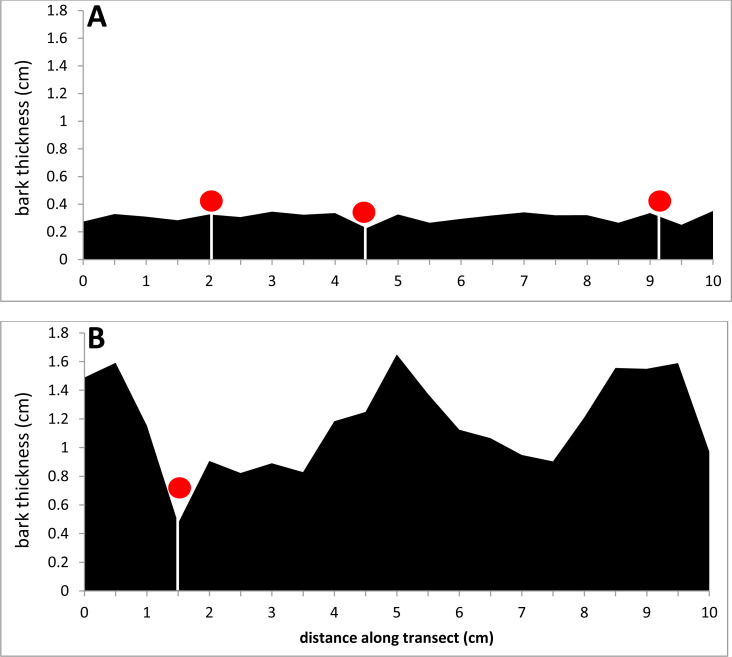
Comparison of two bark samples: in profile. (A) The thin-barked sample #1 had a median bark thickness of 0.26 cm. (B) The thick-barked sample #20 had a median bark thickness of 1.36 cm. KSHB holes are marked with red dots.

**Figure 6:**
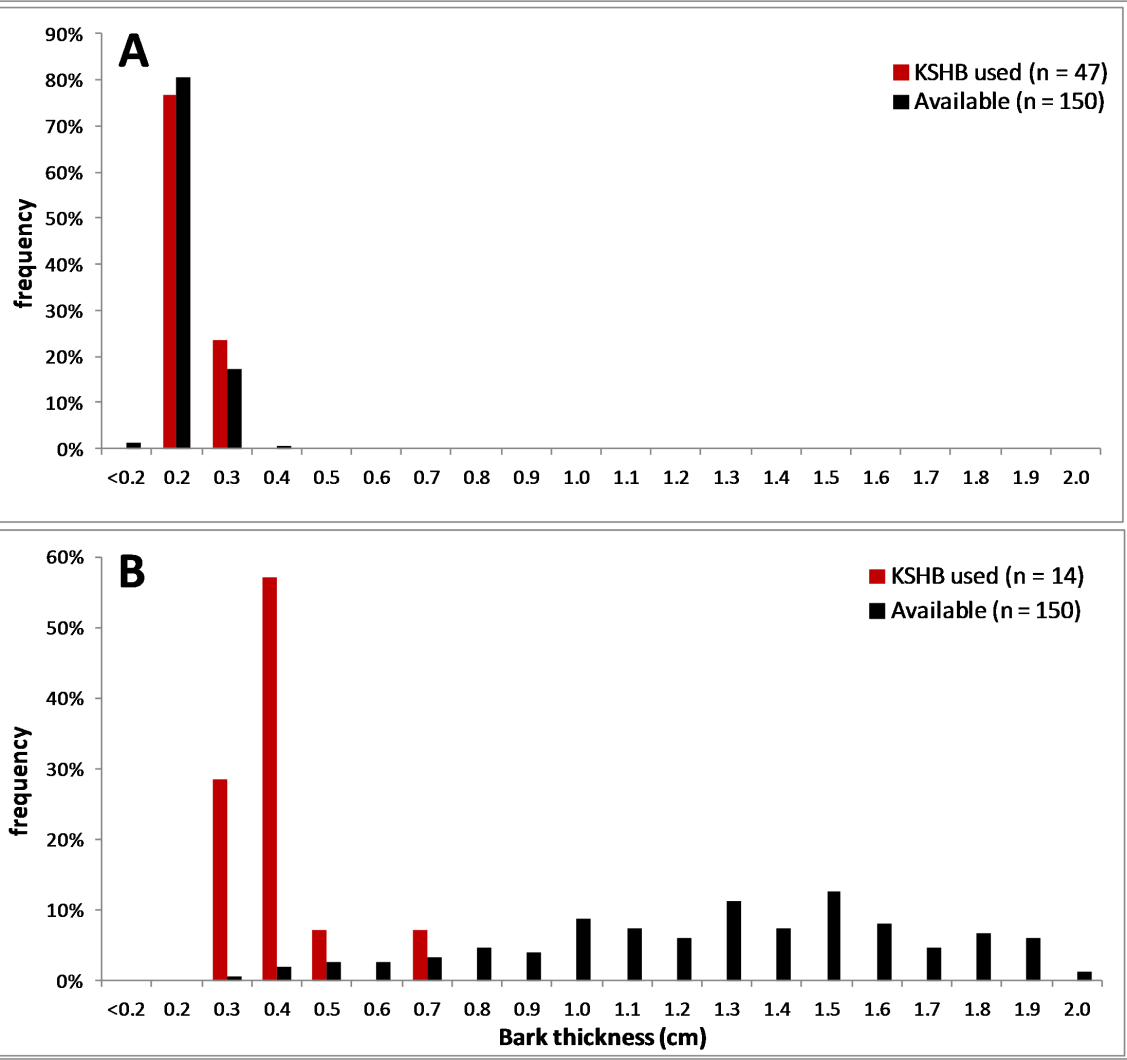
Comparison of two bark samples: thicknesses *used* and *available*. (A) The thin-barked sample #1 had a Proportional Similarity Index of 94%. (B) The thick-barked sample #20 had a Proportional Similarity Index of 9%. The Proportional Similarity Index measures the similarity in the bark thicknesses *used* and *available*.

Two of the 27 bark samples are contrasted here to illustrate the above results (Figs. 4, 5 and 6). An external view shows that the thinner sample had many KSHB holes dispersed over the bark surface, whereas the thicker sample had fewer holes confined to furrows (red pins in Fig. 4). A profile view shows the same dispersion in cross section, i.e., the thinner sample had many KSHB holes dispersed over the bark surface, and the thicker sample had fewer holes confined to furrows, where bark was thinnest (red dots in Fig. 5). The similarity between the frequency distributions of thicknesses *available* and *used* was high in the thinner sample (PSI = 94%) indicating the random distribution of KSHB holes, whereas the similarity was low in the thicker sample (PSI = 9%) indicating the non-random distribution of holes and site selection on the part of the KSHB (Fig. 6). As these results show and as these two samples illustrate, bark thickness influenced both KSHB attack densities and attack locations, i.e., the KSHB bored abundantly through thin bark and avoided boring through thick bark.

### Bark thickness influences KSHB impacts (H2)

**Figure 7:**
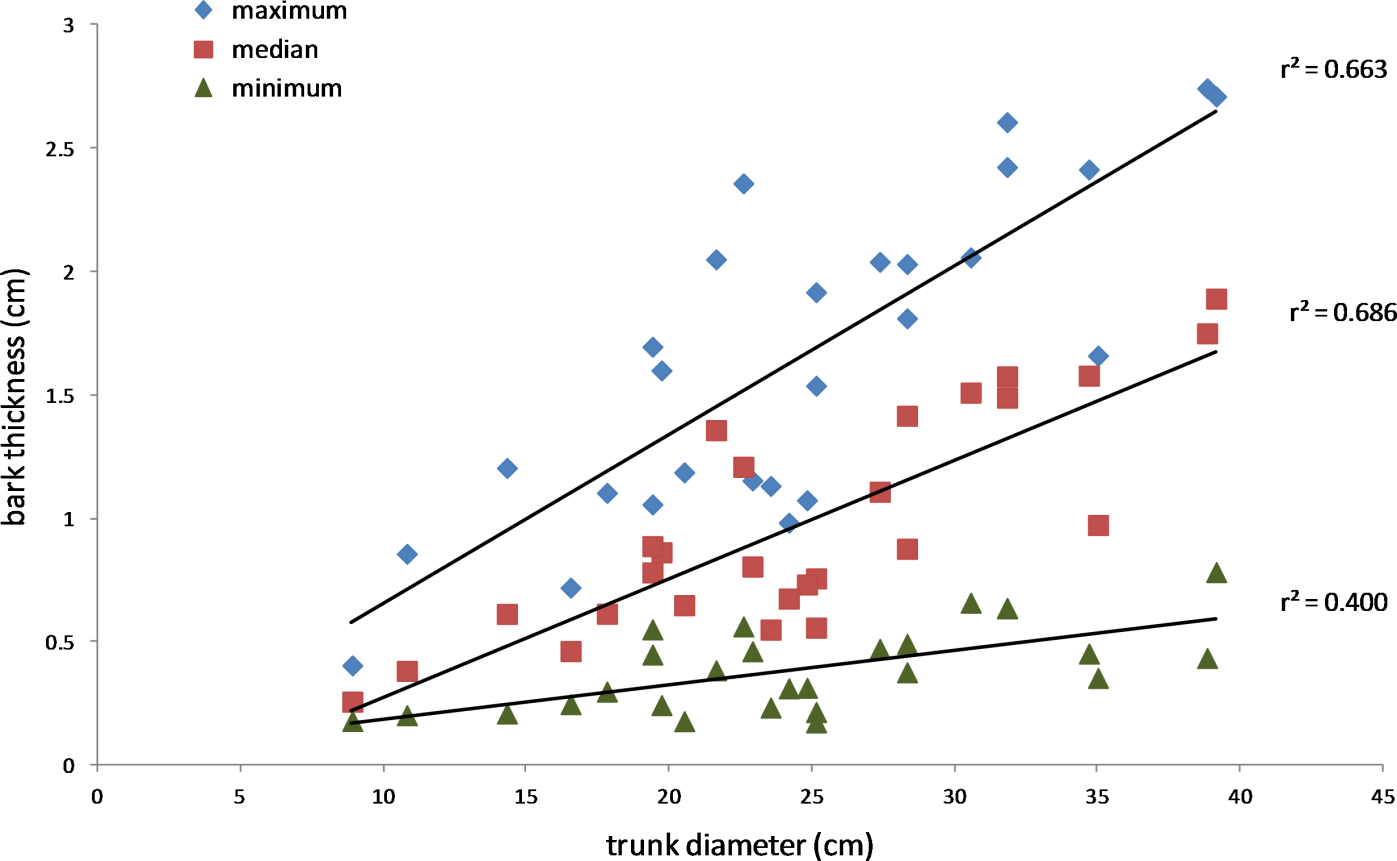
Relationship between bark thickness and trunk diameter. Three measures of *Salix gooddingii* bark thickness are shown for each sample: maximum, median, and minimum thickness. The lines of best fit were produced by linear regression (the *r*^2^ values are shown, and *p* < 0.01, *n* = 27 for each line).

**Figure 8:**
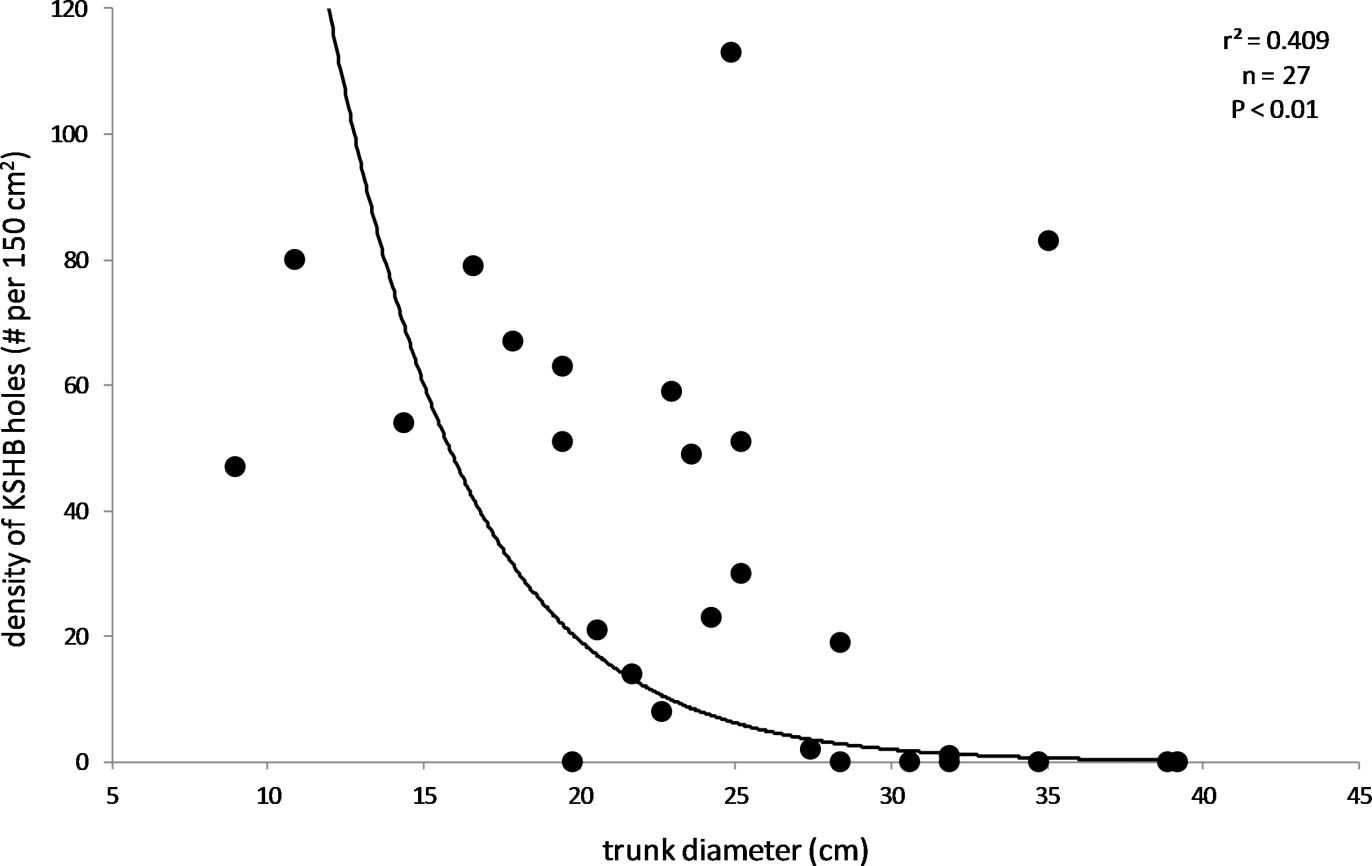
Relationship between density of KSHB holes and trunk diameter. Trunk diameters and hole densities are from the 27 bark samples. The line of best fit was produced by exponential regression (*r*^2^ = 0.409; *p* < 0.01; *n* = 27).

**Figure 9:**
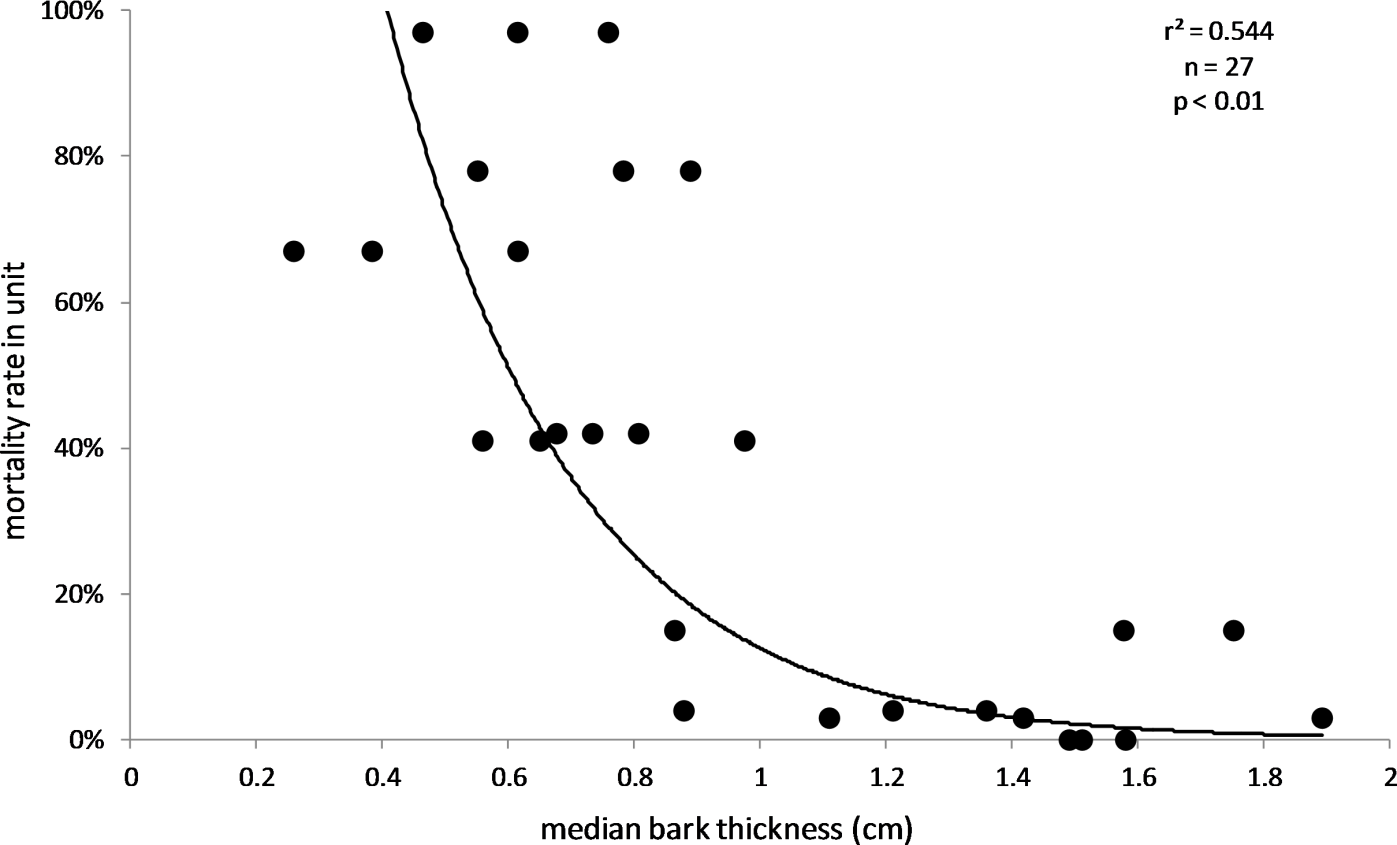
Influence of bark thickness on willow mortality rate. Median bark thicknesses are from the 27 bark samples (collected 2016–2017), and willow mortality rates covered the period 2015–2018 (Boland, 2019). The line and values of p and r^2^ are from exponential regression (*r*^2^ = 0.544; *p* < 0.01; *n* = 27).

The results also supported the hypothesis that bark thickness influences KSHB impacts. First, trunk diameters of the 27 sampled trees ranged from 8.9 to 39.2 cm (Table 1), and, as expected, all measures of bark thickness were positively correlated with trunk diameter, i.e., median bark thickness increased significantly with increasing diameter (*r*^2^ = 0.686, *n* = 27, *p* < 0.01), as did maximum (*r*^2^ = 0.663, *n* = 27, *p* < 0.01) and minimum (*r*^2^ = 0.400, *n* = 27, *p* < 0.01) bark thicknesses (Fig. 7). Second, the density of KSHB holes was negatively correlated with trunk diameter, i.e., hole density decreased significantly with increasing trunk diameter (*r*^2^ = 0.409, *n* = 27, *p* < 0.01; Fig. 8). Third, willow tree mortality rates in the nine units from which the samples were collected ranged from 0 to 97%, and those mortality rates were negatively correlated with bark thickness, i.e., mortality rates decreased significantly as median bark thickness increased (*r*^2^ = 0.544, *n* = 27, *p* < 0.01; Fig. 9). In summary, *S. gooddingii* trees with thick bark had larger diameters, fewer holes per unit area, and lower mortality rates, all consistent with the hypothesis that bark thickness influences KSHB impacts such that KSHB causes more damage to smaller, thinner-barked trees than to larger, thicker-barked ones.

Finally, the mean bark thickness of samples collected from the Wet Forest units was 0.67 cm (± 0.19 cm; *n* = 15) whereas the mean bark thickness of samples collected from Dry Forest units was 1.39 cm (± 0.28 cm; *n* = 12). These bark thicknesses were significantly different (*t*-test, *t* = 7.93, *p* < 0.01), indicating that the more vulnerable, thin-barked trees were significantly more common in the Wet Forests.

### Comparison of two fallen trees

**Figure 10:**
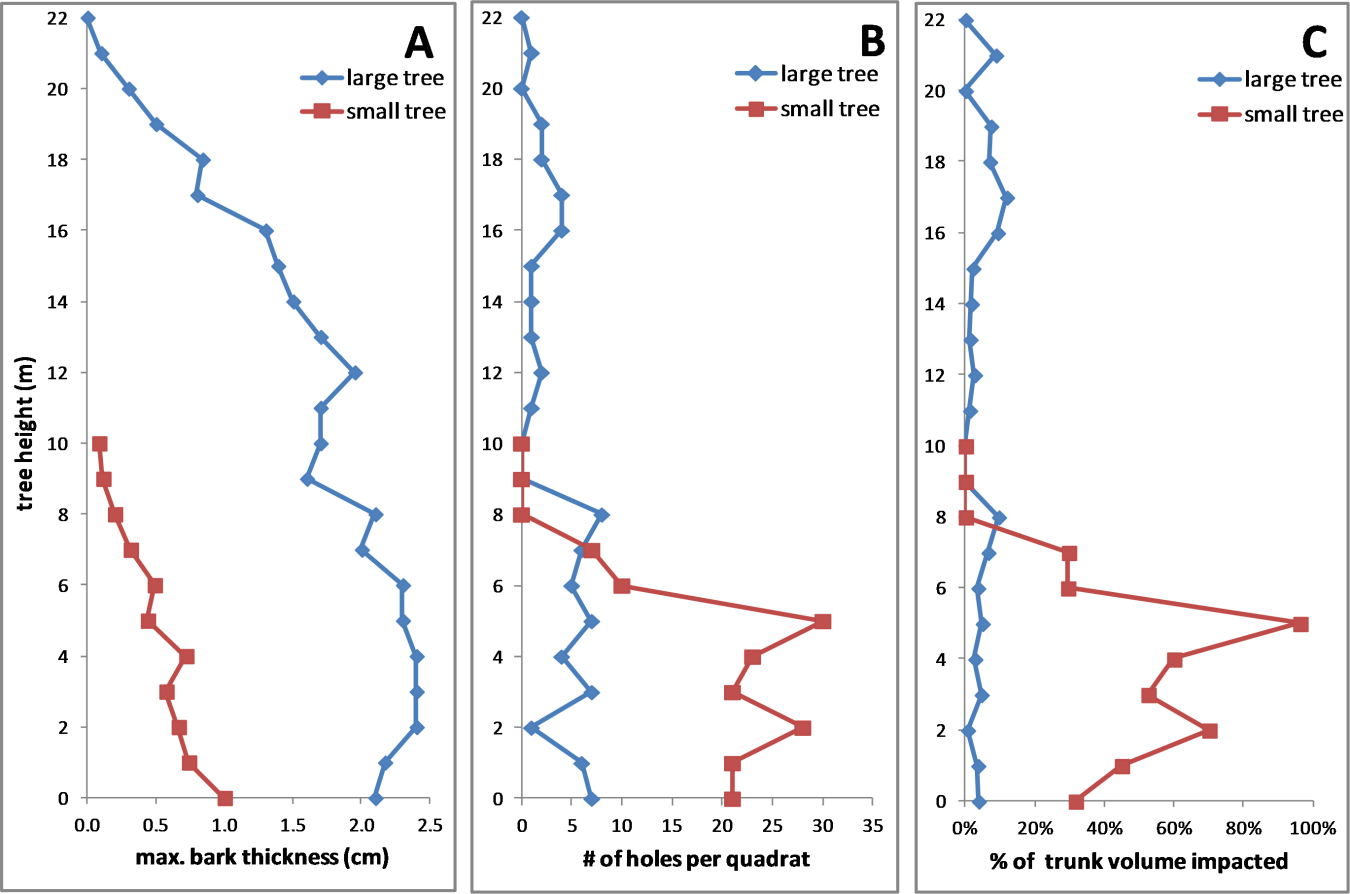
Comparisons of two fallen *Salix gooddingii* trees along their entire trunks. (A) Maximum bark thickness. (B) Number of KSHB holes per quadrat (40 cm^2^). (C) Estimated percent of trunk volume impacted by KSHB. Trunk diameters at breast height were 40.7 cm and 10.8 cm.

In both fallen trees, the maximum bark thickness was greatest on the lower trunk and declined upward (Fig. 10A), the density of KSHB holes was greatest on the lower trunk and declined upwards (Fig. 10B), and the vertical range of infestation was from ground level to about 8 m. Over this range of infestation, the larger tree had considerably thicker bark than the smaller tree (Fig. 10A). As for impact, the estimates of wood volume impacted by the KSHB were strikingly different in the two trees. In the larger tree, the estimated impact was less than 12% in all sections, whereas in the smaller tree the estimated impact was well over 50% in four of eleven sections and up to 96% in one section (Fig. 10C). The low density of holes in the larger, thicker-barked tree therefore led to negligible impacts, whereas the high density of holes in the smaller, thinner-barked tree led to the near destruction of its xylem within large sections of its trunk. In summary, this comparison of the two fallen trees shows why a KSHB infestation does not kill a thick-barked tree but can kill a thin-barked tree.

## Discussion

This detailed examination of KSHB holes in *S. gooddingii* bark revealed that the KSHB preferentially bores through thin bark and, because of this preference, *S. gooddingii* trees with thin bark had more holes per unit area, more damage per unit volume from KSHB tunneling, and higher rates of mortality than those with thicker bark. The KSHB preference for thin bark occurs at the scale of the bark’s ridges and furrows, but it influences damage and mortality patterns at the larger scales of the tree, the forest unit, and the valley. To our knowledge this is the first study to identify bark thickness as a factor that influences the density of KSHB—or any ambrosia beetle—in its host tree, as well as the first to link bark thickness to rates of host tree mortality. These results are vitally important to understanding the impact of the KSHB in southern California and could assist in predicting the impact of other newly transplanted ambrosia beetles worldwide (Hulcr & Stelinski, 2017).

We found compelling evidence that supports both hypotheses regarding bark thickness and the KSHB. With the first hypothesis—that **bark thickness influences KSHB attack densities and locations**—we concluded that, indeed, the KSHB bores abundantly through thin bark and avoids boring through thick bark. Of the more than 900 KSHB holes in the bark samples, none was in bark thicker than 1.07 cm, even though bark more than twice that thickness was abundantly available. Trees with thin bark had abundant, randomly distributed KSHB holes, whereas trees with thick bark had rare, non-randomly distributed KSHB holes, mostly in furrows.

Exactly why the KSHB avoids boring through thick bark is not known. It may be that boring a greater distance for a longer time would deplete an individual beetle’s energy reserves, or that thick bark is too toxic for a boring insect because it contains more layers of secondary chemicals such as tannins, polyphenols, terpenes, suberin, resins, gums and latex, all of which are common in many tree barks (Thomas, 2014). In addition, because the host tree in this study was a willow, it could be due to the presence in the bark of salicin, a chemical known to deter insects and found exclusively in species of the willow family (Salicaceae; Pasteels & Rowell-Rahier, 1992; Wojtech, 2011). Whatever the reason, the KSHB’s preference for boring through thin bark has important ramifications for the trees they infest.

The fact that KSHB holes are not randomly distributed on trees with thick bark suggests that dispersing KSHB individuals, upon landing, actively search for suitable sites in which to initiate an entrance hole. In the Tijuana River Valley, KSHB individuals have occasionally been observed roaming on the bark of willows in what appeared to be searching behavior; in one case a female traveled five cm in eight min, and spent most of the distance in a furrow (Boland personal observation). Searching behavior has also been reported in other ambrosia beetles, i.e., the tea shot hole borer, *Euwallacea fornicatus* (Kalshoven, 1958) and the redbay ambrosia beetle, *Xyleborus glabratus* (Carrillo et al., 2015). Among bark beetles, searching has been reported in the southern pine beetle, *Dendroctonus frontalis*, which, upon landing on a suitable tree, searches for “*an average of 10.2 min and travels 22 cm of bark surface before initiating a gallery entrance, usually within a crevice in the bark*” (p 32; Sullivan, 2011). Our data suggest that KSHB individuals exhibit similar behavior on thick-barked trees and actively search for a suitably thin spot in which to bore.

As for the second hypothesis—that **bark thickness influences KSHB impacts**—we found *S. gooddingii* trees with thick bark had larger diameters, fewer holes per unit area, and lower mortality rates. We conclude that thick bark protects a tree by limiting KSHB hole numbers to survivable low densities by virtue of the limited area of suitably thin bark. Limiting KSHB density is key because it limits the degree of internal damage to xylem tissue and increases the chance of tree survival. *Salix gooddingii* trees can survive a sparse KSHB attack but are killed by an abundant ‘mass accumulation’ (Boland, 2017; Coleman et al., 2019). In the Tijuana River Valley, tagged willows with heavy KSHB infestations died, while those with light to moderate KSHB infestations healed by growing over old KSHB holes (Boland, 2019) or, if the trunk was snapped, by producing large, vigorous resprouts from the infested trunk base (Boland & Uyeda, 2020).

That trees can, in fact, recover from a KSHB infestation indicates that the KSHB’s fungal symbionts are non-pathogenic or only moderately so. In this regard the KSHB is similar to the ambrosia beetle *Platypus quercivorus*, which carries the fungus *Raffaelea quercivora* and causes Japanese oak wilt in oak trees (*Quercus* spp.; Takahashi, Matsushita & Hogetsu, 2010). Like *S. gooddingii*, oaks can survive a sparse *P. quercivorus* attack but are severely damaged or killed by a mass accumulation (Takahashi, Matsushita & Hogetsu, 2010). Similarly, trees attacked by the ambrosia beetle *E. fornicatus* “*only suffer a serious die-back or are killed…when they have been attacked simultaneously by a large number of borers…resulting in…a total interruption of the sap-flow*” (p 153; Kalshoven, 1958). These three species, KSHB, *P. quercivorus* and *E. fornicatus*, are all in stark contrast to the redbay ambrosia beetle, *X. glabratus*, which can cause the death of a host tree with only a few beetles due to its highly virulent fungal symbiont (*Raffaelea lauricola*; Fraedrich et al., 2008; Maner, Hanula & Braman, 2013). While thick bark can protect trees from ambrosia beetles that carry less virulent fungi by reducing the beetle’s attack density, thick bark probably will not protect trees from an attack by ambrosia beetles that carry highly virulent fungi because furrowed thick bark cannot reduce the attack density to zero.

Examination of the two fallen *S. gooddingii* trees, both of which had been infested with KSHB while alive and standing, revealed that the vertical range of KSHB infestation was from the ground to about 8 m. This confirmed that our bark samples taken at breast height on standing *S. gooddingii* trees were within the vertical range of KSHB infestation and were therefore representative. Our calculated estimates of internal damage due to the KSHB along the full trunk found far more extensive damage to the xylem of the smaller, thin-barked tree than of the larger, thicker-barked tree. As the famous double saw-cut experiment has shown, water can ascend a trunk when there is some damage to the xylem, but water conduction is disabled when damage is extensive (Thomas, 2014). We conclude that a mass accumulation of KSHB on thin-barked trees results in tree death due to extensive tunneling within the trunk and extensive damage to the xylem. Conversely, thick-barked trees can survive the limited tunneling and damage caused by low KSHB hole densities.

An important consequence of the KSHB preference for thin bark and avoidance of thick bark is that it gives *S. gooddingii* a refuge in size. Large, mature *S. gooddingii* are more likely to survive a KSHB infestation due to their thick bark, and this explains why many large willows (called “Big Trees” in Boland & Uyeda, 2020) remained standing alive in the Tijuana River Valley in the wake of the KSHB outbreak, while smaller willows in nearby units were severely damaged or killed. Similar refuges in size are likely for other ambrosia beetle hosts, e.g., oaks infested with *P. quercivorus,* but so far have not been reported. The refuge in size seen here in the KSHB-*S. gooddingii* interaction is opposite to some bark beetle-host interactions. For instance, the mountain pine beetle (*Dendroctonus ponderosae),* a bark beetle that infests pines in western North America (Ferrenberg & Mitton, 2014), avoids thin-barked pines and targets thick-barked trees possibly because these bark beetles consume and build their galleries in the inner bark layer (Safranyik & Vithayasai 1971). The refuge in size seen in *S. gooddingii*, is ecologically similar to the well-documented role thick bark plays in protecting mature forest trees from severe wildfire damage (e.g., Ryan, Peterson & Reinhart, 1988).

Bark is a tree’s first line of defense against insects and that defense takes two basic forms—chemical and morphological (Carmona, Lajeunesse & Johnson, 2011). So far entomologists have focused on the chemical defenses (e.g., Bennett & Wallsgrove, 1994; Keeling & Bohlmann, 2006), and the morphological defenses of trees have been largely “*overlooked in studies of forest insects*” (p 843; Ferrenberg & Mitton, 2014). An interesting exception is a study of the influence of bark texture on the attack locations of the mountain pine beetle (*D. ponderosae*) feeding on limber pines (*Pinus flexilis*); that study found more attacks on rough than on smooth bark surfaces because the bark beetles were less able to grip smooth bark and stay on the tree long enough to start a tunnel (Ferrenberg & Mitton, 2014). For the KSHB on *S. gooddingii*, it is the bark thickness that matters, and differences in bark thicknesses can be small but consequential. We found that bark thinner than 0.8 cm was abundantly bored whereas bark thicker than 1.07 cm was never bored, so a difference of only 0.27 cm in bark thickness can be the difference between a heavy, damaging attack and none at all.

This study of bark thickness and the earlier study of nutrient enrichment (Boland & Woodward, 2019) underscore the fact that—within a single species—host tree condition varies from site to site, and the KSHB responds to that variability and causes differing degrees of impact at different sites. This means that to accurately predict the spread and impact of the KSHB, or the closely related *E. whitfordiodendrus*, site characteristics and host tree condition must be incorporated into the predictive models. So far, just one model has been used to predict the impact of these two invasive ambrosia beetles in southern California (McPherson et al., 2017), and it estimated that 11.6 million trees could be lost from the urban forests at a replacement cost of more than $15 billion. Unfortunately the model had many shortcomings: it assumed a high number of host tree species (55 species), assumed high mortality rates for all host species (50%), assumed all sites to be equal, and assumed all host individuals to be equal. Because of these shortcomings this model should be considered a rough first step that greatly exaggerates the likely future impact of the two beetle species. More accurate and rigorous models that include site and tree conditions need to be developed to better predict the impact of these and other new invasives so that the limited funding available for management can be best allocated.

As David Tilman has said, “*the central goal of ecology is to understand the causes of the patterns we observe in the natural world*” (p 3; Tilman, 1988). Here, in this study, we set out to understand the causes of the spatial pattern in the KSHB impact in the Tijuana River Valley, where rates of KSHB-induced damage and mortality were significantly higher in willows near the main river channel than in those farther away (Boland, 2016; Boland & Woodward, 2019). This spatial pattern can be summarized by the case fatality rate, which is the number of willow deaths due to the KSHB divided by the number of willows infested with KSHB; the Wet Forest units combined had a case fatality rate of 39%, whereas the Dry Forest units combined had a case fatality rate of only 9% (Boland & Uyeda, 2020). Our results can explain this spatial pattern in fatality rates because KSHB impacts were more severe in thin-barked trees, and thin-barked trees were more common in the Wet Forests.

Thin bark is a necessary but not sufficient condition for a willow to be fatally infested with KSHB. Bark thickness is one of a suite of key site and tree characteristics that determine whether the impact of a KSHB infestation will be severe or mild (Table 2). The most susceptible site is wet and nutrient enriched, and the most susceptible trees at such a site are the young, fast growing trees with thin bark (allowing the KSHB abundant access) and with wood of low density and high moisture content (providing ideal internal conditions for the KSHB and its associated fungi). All of these conditions occurred in the Wet Forests of the Tijuana River Valley in 2015, leading to the unprecedented KSHB-induced impacts seen in those forests (Boland, 2016; Boland & Woodward, 2019). To date, no other natural site in San Diego County has been so severely affected by the KSHB—and we predict that no other natural site will be so severely affected—because other natural sites lack one or more of these key characteristics (Boland & Woodward, 2019). Again, a KSHB infestation can be likened to a wildfire because the severity of each depends on several factors. The factors that promote a devastating wildfire include certain site conditions (high fuel load) and weather conditions (high air temperature, low relative humidity and high wind velocity), and those that promote a severe KSHB impact include certain site conditions (plenty of water and high nutrient load) and tree conditions (fast growth, thin bark, and wood of low density and high moisture content).

**Table 2:** Characteristics of trees most and least susceptible to the KSHB. Environmental and tree conditions that affect the severity of KSHB impacts and examples of sites. The most susceptible trees suffer high KSHB hole densities and high mortality, whereas the least susceptible trees experience low KSHB hole densities and low mortality. CFR = case fatality rate, i.e., the number of willow deaths due to the KSHB divided by the total number of willows infested with KSHB.

Characteristic	MOST SUSCEPTIBLE	LEAST SUSCEPTIBLE	Source
**ENVIRONMENT**			
distance to water	close (wet)	far (dry)	Boland and Woodward 2019
nutrient enrichment (e.g., sewage)	high	normal	Boland and Woodward 2019
**WILLOW TREES**			
trunk size	small - medium (young)	medium - large (old)	this paper
growth rate	fast	slow	Boland 2018
bark thickness	thin (median <0.8 cm)	thick (median >0.8 cm)	this paper
wood density	low	high	Boland and Woodward 2019
wood moisture content	high	low	Boland and Woodward 2019
**EXAMPLES of SITES**			
In San Diego County	Tijuana River Valley	most other sites	Boland and Woodward 2019
In Tijuana River Valley (CFR)	Wet Forest units (39%)	Dry Forest units (9%)	Boland and Uyeda 2020

In a recent review of ambrosia beetle symbiosis, Hulcr & Stelinski (2017, page 296) stated: “*in ambrosia beetle research, the role of the environment and preexisting conditions of the trees has not yet been well appreciated, even though it appears to determine the impact of these beetles.*” In 2017, therefore, links between the environment, tree condition, and shot hole borer impact had been suspected but not yet identified. For KSHB and willows, we think we have identified the environmental and preexisting conditions of the trees that ultimately determine the severity of a KSHB attack (Table 2), and thickness of bark is one of the most important.

##  Supplemental Information

10.7717/peerj.10755/supp-1 Supplemental Information 1 All bark thickness data. Each figure has its own data sheet.

10.7717/peerj.10755/supp-2 Supplemental Information 2 Precision measurements. The same bark sample was measured twice to gauge precision. In each trial *n* = 150.

## Additional Information and Declarations

### Field Study Permissions

The following information was supplied relating to field study approvals (i.e., approving body and any reference numbers): The County of San Diego Department of Parks and Recreation and the US Fish and Wildlife Service allowed access to their properties via permits ROE08.04.16 and 19001TJS, respectively.

### Data Availability

The following information was supplied regarding data availability: Raw data are available in the Supplemental Files.
